# HIF-1 maintains a functional relationship between pancreatic cancer cells and stromal fibroblasts by upregulating expression and secretion of Sonic hedgehog

**DOI:** 10.18632/oncotarget.24156

**Published:** 2018-01-11

**Authors:** Tomohiro Katagiri, Minoru Kobayashi, Michio Yoshimura, Akiyo Morinibu, Satoshi Itasaka, Masahiro Hiraoka, Hiroshi Harada

**Affiliations:** ^1^ Department of Radiation Oncology and Image-Applied Therapy, Kyoto University Graduate School of Medicine, Sakyo-Ku, Kyoto 606-8507, Japan; ^2^ Laboratory of Cancer Cell Biology, Department of Genome Dynamics, Radiation Biology Center, Kyoto University, Yoshida Konoe-Cho, Sakyo-Ku, Kyoto 606-8501, Japan; ^3^ Precursory Research for Embryonic Science and Technology, Japan Science and Technology Agency (JST), Kawaguchi, Saitama 332-0012, Japan

**Keywords:** tumor hypoxia, hypoxia-inducible factor 1 (HIF-1), Sonic hedgehog signaling pathway, stromal fibroblasts, pancreatic cancers

## Abstract

Hypoxic and stroma-rich microenvironments, characteristic features of pancreatic cancers, are strongly associated with a poor prognosis. However, whether and how hypoxia increases stromal compartments remain largely unknown. Here, we investigated the potential importance of a master regulator of the cellular adaptive response to hypoxia, hypoxia-inducible factor-1 (HIF-1), in the formation of stroma-rich microenvironments of pancreatic tumors. We found that pancreatic cancer cells secreted more Sonic hedgehog protein (SHH) under hypoxia by upregulating its expression and efficiency of secretion in a HIF-1-dependent manner. Recombinant SHH, which was confirmed to activate the hedgehog signaling pathway, accelerated the growth of fibroblasts in a dose-dependent manner. The SHH protein secreted from pancreatic cancer cells under hypoxic conditions promoted the growth of fibroblasts by stimulating their Sonic hedgehog signaling pathway. These results suggest that the increased secretion of SHH by HIF-1 is potentially responsible for the formation of detrimental and stroma-rich microenvironments in pancreatic cancers, therefore providing a rational basis to target it in cancer therapy.

## INTRODUCTION

Pancreatic cancer is a deadly disease because it is highly resistant to conventional therapies, such as chemotherapy and radiation therapy [1]. Despite advances in treatment strategies, pancreatic cancer patients have a dismal prognosis, with 5-year overall survival rates of about 0-6% [2, 3]. Characteristic features of pancreatic cancer strongly associated with the poor prognoses of patients and therapeutic resistance are the existence of both hypoxic regions and stroma-rich microenvironments [4].

It has been established that the partial pressure of oxygen is spatially heterogeneous in malignant solid tumors due to the imbalance between the supply of oxygen and its consumption by cancer cells and due to the limited distance molecular oxygen can diffuse from blood vessels in such tumors [5–7]. Hypoxic regions, where cancer cells are under low-oxygen conditions below physiological levels, are known to chronically arise at 70-100 μm from functional blood vessels [5–7]. The intratumoral volume of hypoxic regions has been reported to be positively correlated with the poor prognosis of pancreatic cancer patients [8].

Accumulating evidence has suggested that a factor associated with the poor prognosis as well as malignant progression of pancreatic cancers is hypoxia-inducible factor 1 (HIF-1) [9, 10]. HIF-1 is a heterodimeric transcription factor that is composed of an ɑ-subunit (HIF-1α) and β-subunit (HIF-1β) [11], and its activity is mainly regulated through oxygen-dependent changes in protein stability and the transactivation activity of HIF-1α [12, 13]. Once HIF-1α becomes stabilized and activated under hypoxic conditions, it, in combination with HIF-1β, induces the expression of hundreds of genes responsible for malignant cancer progression [12]. Although the positive correlations between HIF-1α expression levels as well as the volume of hypoxic regions and both the poor prognosis of pancreatic cancer patients [9, 10] and decreased anti-tumor effects of HIF-1α-targeting drugs in pancreatic tumors [14, 15] have been repeatedly reported, key molecular mechanisms behind them are still unclear.

Another characteristic feature of pancreatic cancers is the stroma-rich microenvironment, which has been reported to result from the activation of the Sonic hedgehog signaling pathway, aberrant proliferation of fibroblasts, and overproduction of extracellular matrix (ECM) [16]. Specifically, the mature form of Sonic hedgehog protein (SHH) is secreted from pancreatic cancer cells after removal of the signal peptide and autocatalytic processing [17]. The secreted SHH protein then cancels the negative regulation of smoothened (SMO) by patched (PTCH) through the direct binding of SHH to PTCH on the surface of fibroblasts, leading to the activation of a transcription factor, Gli-1, in fibroblasts. Because Gli-1 has an activity to upregulate cellular proliferation, differentiation, and survival by inducing the expressions of target genes, such as *cyclin D1, c-myc, bcl2*, and *snail*, the paracrine signaling is thought to be important in the formation of the stroma-rich microenvironment of pancreatic cancers. Thus, marked efforts have been devoted to clarify the characteristic features of each hypoxic condition and the stroma-rich microenvironment in pancreatic cancers; however, whether and how HIF-1 and the Sonic hedgehog signaling pathway influence each other and eventually create the pancreatic cancer-distinctive microenvironments have yet to be fully elucidated.

In the present study, we investigated the functional and mechanistic linkage between HIF-1 and Sonic hedgehog signaling to better understand whether and how the stroma-rich microenvironment arises in pancreatic cancers. We revealed that pancreatic cancer cells secrete more SHH under hypoxic conditions by increasing the efficiency of secretion as well as expression of SHH in a HIF-1-dependent manner, and promote the growth of fibroblast cells by stimulating the hedgehog signaling pathway in a paracrine manner.

## RESULTS

### Increase in the efficiency of secretion as well as expression of SHH under hypoxia in a HIF-1-dependent manner

**Figure 1:**
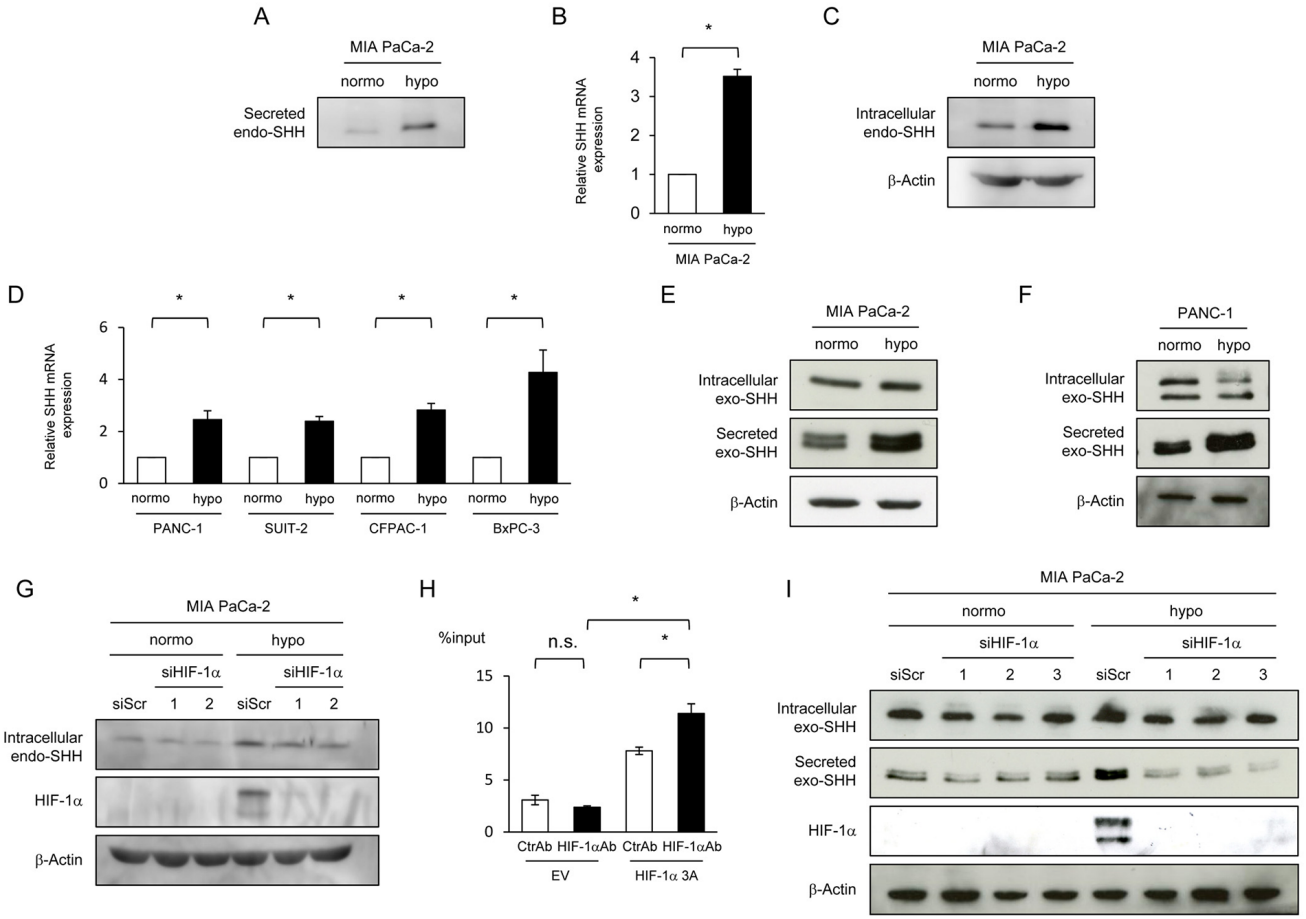
HIF-1-dependent increases in the expression and efficiency of secretion of SHH proteins under hypoxic conditions **(A-D)** The indicated cells were cultured under normoxic or hypoxic conditions, and culture media, total RNA extracts, and cell lysates were subjected to Western blotting for Secreted SHH (A), qRT-PCR for SHH mRNA (B, D), and Western blotting for intracellular SHH (C), respectively. **(E, F)** After the indicated cells were transiently transfected with pcDNA4/SHH and cultured under normoxic or hypoxic conditions, cell lysate and culture media were harvested and subjected to Western blotting for intracellular SHH (top), Secreted SHH (middle), and β-actin (bottom). **(G)** MIA PaCa-2 cells were cultured under normoxic or hypoxic conditions with (siHIF-1α 1 or 2) or without (siScr) silencing the *hif-1α* gene, and cell lysates were subjected to Western blotting for the indicated proteins. **(H)** Hela cells were transiently transfected with the pGL3/SHH promoter plasmid and with either pcDNA4/HIF-1α 3A or pcDNA4/*myc*-His A, and the resultant cell lysates were subjected to an IP experiment using either anti-HIF-1α antibody (HIF-1αAb) or its isotype control (CtrlAb) coupled with a quantitative RT-PCR experiment using the specific primers to the SHH promoter. **(I)** MIA PaCa-2 cells were treated with the same conditions as G in parallel with the transient transfection with pcDNA4/SHH, and the resultant cell lysates and culture media were subjected to Western blotting for intracellular Exo SHH (top), secreted exo SHH (the 2^nd^ from the top), HIF-1α (the 3^rd^ from the top), and β-actin (bottom).

To examine whether the amount of SHH proteins secreted from cancer cells is increased under hypoxic conditions, we cultured pancreatic cancer cells, MIA PaCa-2, under normoxic or hypoxic conditions, and harvested culture media for Western blot analysis. Because the amount of SHH protein secreted from the endogenous *shh* gene into the culture media was below detectable levels, we quantitatively concentrated the culture media using a centrifugal filter (*see* Materials and Methods for details). Western blot analysis revealed that the amount of secreted SHH proteins was markedly increased under hypoxic conditions (Figure 1A). Because it was unclear which mechanism was responsible for the increase, a hypoxia-mediated induction of SHH expression or hypoxia-mediated increase in SHH secretion efficiency, or both, we first analyzed the influence of hypoxic stimuli on the expression levels of the SHH gene. Quantitative real-time PCR (qRT-PCR) revealed an increase in SHH mRNA levels under hypoxic conditions, resulting in the elevated expression levels of the SHH protein (Figure 1B, 1C). Hypoxic induction of the SHH gene expression was observed in other pancreatic cancer cell lines, such as PANC-1, SUIT-2, CFPAC-1, and BxPC-3 (Figure 1D). These results indicate that the induction of SHH gene expression was, at least in part, involved in the increased amount of SHH proteins secreted from pancreatic cancer cells under hypoxia.

We subsequently tested whether the efficiency of SHH secretion was also enhanced by hypoxic stimuli and involved in the increase in secreted SHH protein under hypoxic conditions. To specifically focus on the efficiency of SHH secretion, we transiently transfected MIA PaCa-2 cells with the plasmid pcDNA4/SHH, which expresses detectable levels of the SHH gene from the constitutively active cytomegalovirus (CMV) promoter. Western blot analysis using culture media demonstrated that the cells secreted more SHH when exposed to hypoxic conditions (*See* Secreted exo-SHH in Figure 1E); meanwhile, they expressed almost equivalent levels of SHH regardless of oxygen conditions (*See* Intracellular exo-SHH in Figure 1E). The same result was observed in another pancreatic cancer cell line, PANC-1 (Figure 1F). Taken together, these results indicate that both the expression and secretion of SHH were upregulated in pancreatic cancer cell lines under hypoxic conditions.

Because HIF-1 is a transcription factor that plays key roles in cellular adaptive responses to hypoxia, we hypothesized that it might be involved in the hypoxia-dependent induction of SHH expression and secretion. To directly investigate whether HIF-1 mediates the increase in SHH expression by hypoxic treatment, we cultured MIA PaCa-2 cells with or without silencing the endogenous HIF-1α gene under normoxic or hypoxic conditions, and harvested their cell lysates for Western blot analysis. The HIF-1α knockdown slightly but significantly suppressed the induction of SHH expression under hypoxic conditions (Figure 1G). Moreover, an immunoprecipitation (IP) experiment using anti-HIF-1α antibody followed by a real-time PCR experiment using DNA primers specific to the promoter region of the SHH gene demonstrated that HIF-1 specifically bound to the SHH promoter region (Figure 1H). These results are compatible with a previous study showing that HIF-1 is involved in the expression of SHH [3]. To examine whether HIF-1 is also involved in the hypoxia-mediated increase in the efficiency of SHH secretion, we introduced HIF-1α siRNA into MiaPaCa-2 cells. Knockdown of the HIF-1 ɑ gene did not influence the expression levels of the exogenous SHH protein expressed by the SHH expression vector, but markedly inhibited the hypoxia-dependent increase in the amount of SHH protein secreted into the culture media (Figure 1I). These results directly indicate that HIF-1 played important roles in the upregulation of both the expression and secretion of SHH.

### Hypoxia-mediated increase in SHH secreted from pancreatic cancer cells upregulates hedgehog signaling activity in fibroblasts

**Figure 2:**
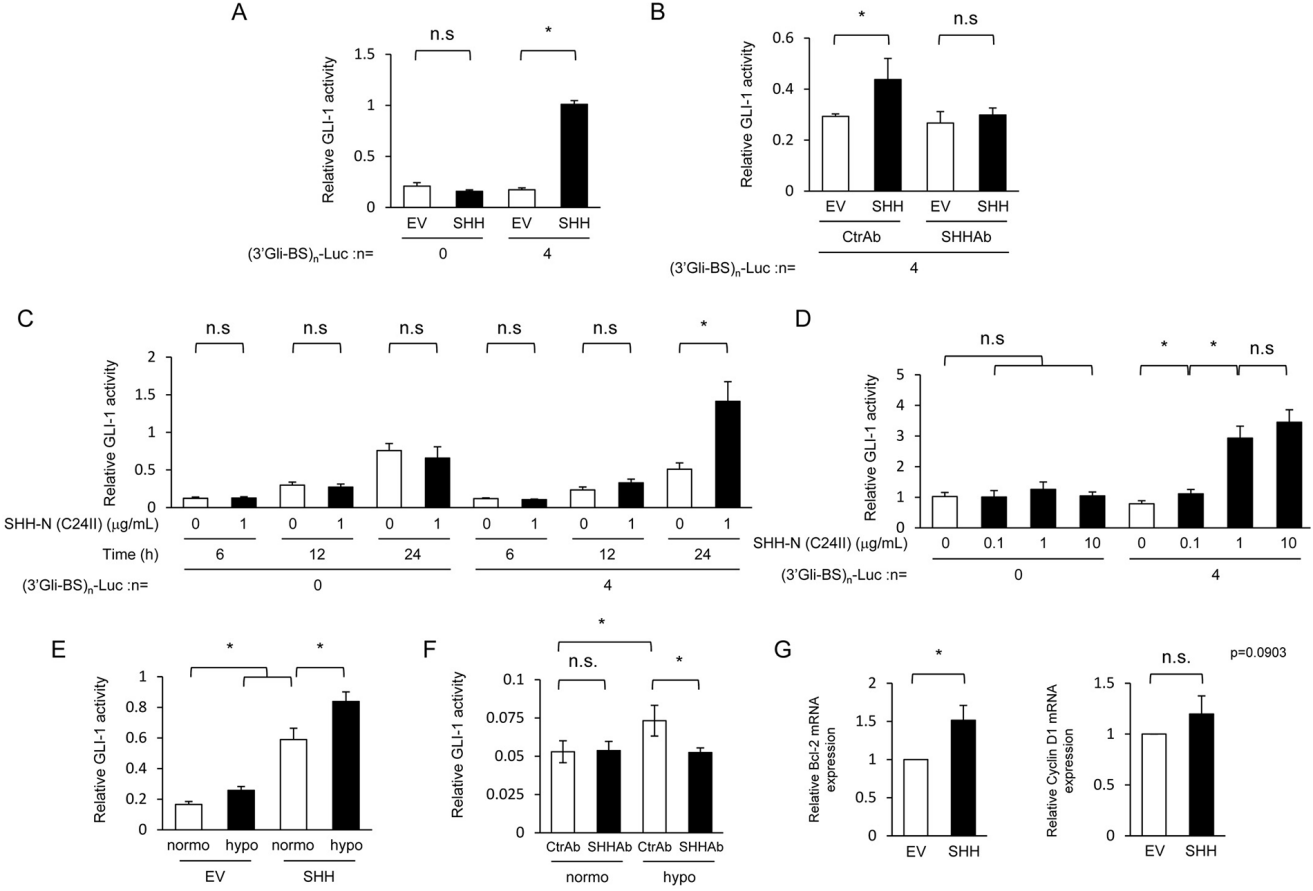
Hypoxia-dependent increase in SHH secreted from pancreatic cancer cells upregulates hedgehog signaling activity in fibroblasts **(A)** After being transiently transfected with either pGL3/(3’Gli-BS)_0_-Luc or pGL3/(3’Gli-BS)_4_-Luc, MEFs were cultured for 24 h in the conditioned medium harvested from MIA PaCa-2 cells transiently transfected with either pcDNA4/SHH (SHH) or pcDNA4/*myc*-His A, and subjected to the luciferase assay. **(B)** After being transiently transfected with pGL3/(3’Gli-BS)_4_-Luc, MEFs were cultured for 48 h in either of the conditioned media harvested under the same conditions as A in the presence of either SHH-neutralizing antibody (SHHAb) or control antibody (CtrAb) (1 μg/mL), and subjected to the luciferase assay. **(C & D)** After being transiently transfected with either pGL3/(3’Gli-BS)_0_-Luc or pGL3/(3’Gli-BS)_4_-Luc, MEFs were treated with the indicated concentrations of the recombinant SHH protein during the indicated durations, and subjected to the luciferase assay. **(E)** After being transiently transfected with pGL3/(3’Gli-BS)_4_-Luc and pcDNA6/SMO, MEFs were cultured for 48 h in the conditioned medium, which had been harvested from culture dishes of MIA PaCa-2 cells transfected with either pcDNA4/SHH (SHH) or pcDNA4/*myc*-His A and cultured for 24 h under normoxic or hypoxic conditions, and subjected to the luciferase assay. **(F)** After being transiently transfected with pGL3/(3’Gli-BS)_4_-Luc, MEFs were cultured for 24 h in the conditioned media harvested from MIA PaCa-2 cells under normoxic or hypoxic conditions in the presence of SHH-neutralizing (SHHAb) or control (CtrAb) antibody (1 μg/mL), and subjected to the luciferase assay. **(G)** MEFs were cultured for 24 h in the conditioned media harvested under the same conditions as A, and total RNA extracts were subjected to qRT-PCR to quantify the levels of bcl-2 and cyclin D1 transcripts.

We next examined the influence of the hypoxia-mediated increase in the amount of secreted SHH protein on the activity of the hedgehog signaling pathway in fibroblasts. SHH is known to activate a key transcription factor, Gli-1, through the inactivation of PTCH and resultant activation of SMO in the hedgehog signaling pathway [16, 17]; therefore, we considered that a reporter gene expressing luciferase bioluminescence in a Gli-1-dependent manner would meet our purpose (Supplementary Figure 1A). Among a series of Gli-1 reporter genes we constructed using 0-12 repeats of the optimized consensus sequence for Gli-1 (3’Gli-Binding Site: 3’Gli-BS) [18], the reporter vector containing 4 repeats of 3’Gli-BS (*(3’Gli-BS)_4_-luc* reporter gene) exhibited significant and sufficient luciferase activity in response to Gli-1 overexpression (Supplementary Figure 1B, 1C). The *(3’Gli-BS)_4_-luc* reporter gene showed a marked increase in luciferase bioluminescence after the forced expression of SMO as well, indicating its usefulness to monitor the hedgehog signaling activity (Supplementary Figure 1D, 1E). To investigate whether SHH proteins secreted from pancreatic cancer cells actually activate the hedgehog signaling pathway in fibroblasts, we transiently transfected MIA PaCa-2 cells with the SHH expression vector or its empty vector, harvested the resultant conditioned media, cultured mouse embryonic fibroblasts (MEFs) in either of them after transient transfection with the *(3’Gli-BS)_4_-luc* reporter gene, and performed the luciferase assay. We confirmed that the culture medium containing secreted SHH proteins increased luciferase bioluminescence in fibroblasts (Figure 2A). SHH-neutralizing antibody abrogated the conditioned medium-induced increase in luciferase activity (Figure 2B). When the fibroblasts were cultured for the indicated durations with various concentrations of a recombinant amino-terminal fragment of SHH (C24II) protein [19], which was purified as shown in Supplementary Figure 2 as an active form of SHH, fibroblasts exhibited significantly higher levels of luciferase bioluminescence from the *(3’Gli-BS)_4_-luc* reporter gene in both time- and dose-dependent manners (Figure 2C, 2D). These results collectively indicate that the SHH-mediated activation of the hedgehog signaling activity in fibroblasts could be specifically monitored using the (3’Gli-BS)_4_-luc reporter gene.

With this useful assay system, we investigated whether pancreatic cancer cells cultured under hypoxic conditions potentially stimulate the hedgehog signaling of fibroblasts *via* the Sonic hedgehog pathway. The conditioned culture medium, which was harvested under hypoxic conditions after the forced expression of SHH as shown in Figure 2A, upregulated the hedgehog signaling in fibroblasts more than that harvested under normoxic conditions (Figure 2E). The conditioned culture medium harvested from MIA PaCa-2 cells under hypoxic conditions as in Figure 1A without transfecting the SHH expression vector upregulated the hedgehog signaling pathway in fibroblasts more than that harvested under normoxic conditions (Figure 2F). The upregulation was completely abrogated in the presence of the SHH-neutralizing antibody (Figure 2F), suggesting that the SHH protein endogenously expressed in and secreted from cancer cells under hypoxic conditions significantly evoked the Sonic hedgehog signaling in fibroblasts in a paracrine manner. Actually, we could confirm, through qRT-PCR analyses, that the conditioned culture medium induced the expressions of the representative downstream factors of the hedgehog signaling, such as *bcl-2* and *cyclin D1* (Figure 2G). These results indicate the existence of paracrine signaling between hypoxic pancreatic cancer cells and fibroblasts *via* the Sonic hedgehog pathway.

### Hypoxia-mediated increase in SHH secreted from pancreatic cancer cells accelerates the growth of fibroblasts *via* activation of the Sonic hedgehog pathway

**Figure 3:**
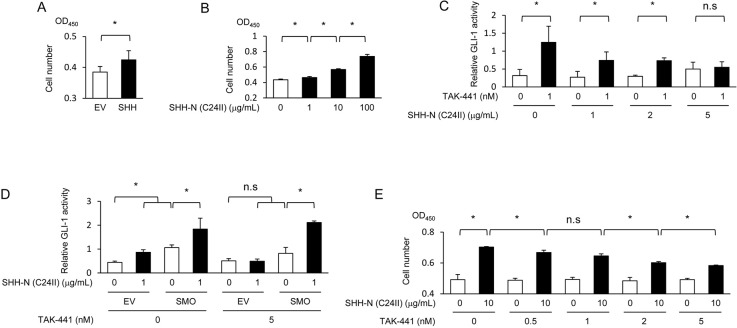
SHH proteins exhibit an ability to enhance the growth of fibroblasts *via* the activation of the Sonic hedgehog pathway **(A)** After being cultured in the conditioned media harvested under the same conditions as in Figure 2A, MEFs were subjected to the cell proliferation assay. **(B)** After being treated with the indicated concentrations of the recombinant SHH protein, MEFs were subjected to the cell proliferation assay. **(C)** After being transiently transfected with pGL3/(3’Gli-BS)_4_-Luc, MEFs were treated with or without the recombinant SHH protein (1 μg/mL) and with the indicated concentrations of a SMO-specific inhibitor, TAK-441, for 24 h, and subjected to the luciferase assay. **(D)** After being transiently co-transfected with pGL3/(3’Gli-BS)_4_-Luc and either pcDNA6/SMO (SMO) or pcDNA6/V5-His A, MEFs were treated with the same conditions as C, and subjected to the luciferase assay. **(E)** MEFs were treated with or without the recombinant SHH protein (10 μg/mL) and with the indicated concentrations of TAK-441, and subjected to the cell proliferation assay.

Next, we investigated the impact of a hypoxia-dependent increase in the amount of secreted SHH protein on the characteristics of fibroblasts. We focused on the influence on the proliferative potential because the activation of the hedgehog signaling was confirmed to induce the expression of the proliferation-related gene *cyclin D1* (Figure 2G). To examine the influence of SHH proteins secreted from pancreatic cancer cells on the growth of fibroblasts through the activation of the Sonic hedgehog pathway, we first cultured fibroblasts in the conditioned medium harvested under the same conditions as in Figure 2A, and performed the cell proliferation assay. We observed that the conditioned medium containing secreted SHH proteins as well as that containing the recombinant SHH protein accelerated the growth of fibroblasts (Figure 3A, 3B). We then treated fibroblasts with various concentrations of a SMO specific inhibitor, TAK-441 [20], and performed the luciferase assay using the Gli-1 reporter gene in order to investigate the involvement of the Sonic hedgehog signaling pathway. As expected, treatment with TAK-441 suppressed the SHH-mediated increase in the hedgehog signaling activity in fibroblasts in a dose-dependent manner (Figure 3C), but the effect of TAK-441 was completely abolished by the overexpression of SMO (Figure 3D). The cell proliferation assay also revealed that treatment with TAK-441 suppressed the SHH-stimulated growth of the fibroblasts as well (Figure 3E). These results collectively indicate that secreted SHH proteins enhance the proliferation of fibroblasts through activation of the Sonic hedgehog pathway.

**Figure 4:**
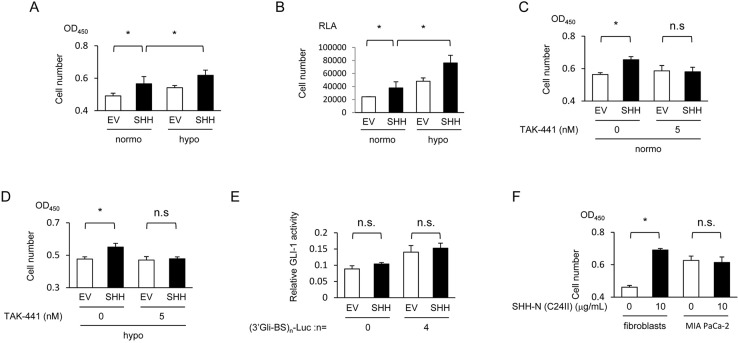
Hypoxia-mediated increase in SHH secreted from pancreatic cancer cells enhances the growth of fibroblasts *via* activation of the Sonic hedgehog pathway **(A)** MEFs were cultured in the conditioned media, which were harvested using the same method as in Figure 3A under normoxic or hypoxic conditions, and subjected to the cell proliferation assay using SF reagent. **(B)** After being transfected with pRL-CMV, MEFs were cocultured with MIA PaCa-2 cells transfected with either pcDNA4/SHH (SHH) or pcDNA4/*myc*-His A under normoxic or hypoxic conditions, and subjected to the cell proliferation assay using Renilla luciferase. RLA=relative luciferase activity. **(C & D)** After being cultured in the conditioned media, which were harvested under the same conditions as in Figure 3A under normoxic (C) or hypoxic (D) conditions, and treated with the indicated concentrations of TAK-441, MEFs were subjected to the cell proliferation assay using SF reagent. **(E)** MIA PaCa-2 cells were transiently co-transfected with either pGL3/(3’Gli-BS)_0_-Luc or pGL3/(3’Gli-BS)_4_-Luc and either pcDNA4/SHH (SHH) or pcDNA4/*myc*-His A, and subjected to the luciferase assay. **(F)** The indicated cells were treated with or without the recombinant SHH protein (10 μg/mL), and subjected to the cell proliferation assay using SF reagent.

We subsequently investigated whether pancreatic cancer cells exposed to hypoxic conditions promote the growth of fibroblasts *via* the activation of Sonic hedgehog signaling. The conditioned culture medium, which was harvested under hypoxic conditions after the forced expression of SHH as in Figure 2A, accelerated the growth of fibroblasts more than that harvested under normoxic conditions (Figure 4A). Growth of fibroblasts was confirmed to be accelerated when they were cocultured with the SHH-expressing MIA PaCa-2 cells (Figure 4B). The positive impact was observed regardless of oxygen conditions, but it was much greater under hypoxic conditions (Figure 4B). The cell proliferation assay revealed that treatment with TAK-441 completely abrogated the accelerated growth of fibroblasts (Figure 4C, 4D). All of these results suggest the important role of paracrine signaling between hypoxic pancreatic cancer cells and fibroblasts in the enhanced growth of fibroblasts through the activation of Sonic hedgehog signaling.

Next, we investigated the possibility that SHH proteins secreted from pancreatic cancer cells activate the Sonic hedgehog pathway in the cancer cells themselves, which in turn would further increase the growth of fibroblasts *via* paracrine signaling. The luciferase assay demonstrated that co-transfection of MIA PaCa-2 cells with the SHH expression vector and *(3’Gli-BS)_4_-luc* reporter vector did not significantly stimulate the hedgehog signaling activity in an autocrine manner (Figure 4E). In addition, the recombinant SHH protein did not stimulate the proliferation of MIA PaCa-2 cells at all (Figure 4F).

## DISCUSSION

In the present study, we examined the mechanistic and functional link between HIF-1 and the Sonic hedgehog signaling pathway to learn how the stroma-rich microenvironments develop in typically hypoxic pancreatic cancer. We revealed that pancreatic cancer cells, when exposed to hypoxic conditions, secreted more SHH by enhancing the efficiency of secretion as well as the expression of SHH in a HIF-1-dependent manner. We also found that the SHH proteins secreted from hypoxic pancreatic cancer cells promoted the growth of fibroblasts by stimulating their Sonic hedgehog signaling pathway. Our findings promote understanding of the important link between the hypoxic regions and stroma-rich microenvironments, which have been strongly associated with poor prognoses of pancreatic cancer patients.

Our results showing that pancreatic cancer cells secreted more SHH under hypoxic conditions by expressing more SHH in a HIF-1-dependent manner are consistent with a previous report [3]. Also, as a novel finding, we found that the efficiency of SHH secretion was also upregulated under hypoxic conditions in a HIF-1-dependent manner, contributing to the increase in the amount of secreted SHH protein under hypoxia. Although the molecular mechanism behind this remains to be elucidated, this is the first study to demonstrate that HIF-1 plays an important role in enhancing the efficiency of SHH secretion under hypoxic conditions.

We demonstrated that SHH proteins secreted from pancreatic cancer cells activated the Sonic hedgehog pathway of fibroblasts and accelerated the proliferation of fibroblasts in a paracrine manner. On the other hand, we could not confirm the so-called autocrine effect on pancreatic cancer cells themselves. Our result is not necessarily consistent with a previous report that the growth of pancreatic cancer cells was enhanced not only when treated with recombinant SHH proteins [21] but also when transiently transfected with an SHH expression vector [22]. The discrepancy might have been caused by the difference in the experimental setting, *e.g.*, serum concentrations (1% in our study but 0.8% in that of Nakashima *et al*. [22]) and cancer cell lines used (MIA PaCa-2 in ours, PANC-1 in Ma *et al*. [21], and SUIT-2 and AsPC-1 in Nakashima *et al*. [22]).

The luciferase assay using the *(3’Gli-BS)_4_-luc* reporter gene demonstrated that the SMO specific inhibitor, TAK-441, completely abolished the SHH-induced hedgehog signaling activity at a dose of 5 nM. On the other hand, the cell proliferation assay showed that the growth of fibroblasts accelerated by SHH was not necessarily suppressed to its basal levels even by the same concentration of TAK-441. These results collectively suggest that the SHH-accelerated growth of fibroblasts was not only promoted by the Sonic hedgehog pathway but was also positively influenced by another pathway.

Here, we demonstrated the possibility that tumor hypoxia, which is a characteristic feature of pancreatic tumor tissues, would contribute to the formation of a stroma-rich microenvironment through the sequential activations of HIF-1 and Sonic hedgehog signaling in cancer cells and fibroblasts, respectively. Meanwhile, previous studies suggested that a stroma-rich microenvironment would make pancreatic tumor tissues hypoxic as increases in stromal components, such as fibroblasts and hyaluronan, reduce tumor perfusion by compressing blood vessels [23, 24]. Taken together, these studies indicate that a positive-feedback loop exists between tumor hypoxia and the stroma-rich microenvironment and this plays an important role in the formation and maintenance of the pancreatic cancer-distinctive microenvironment. It would be interesting to test whether the disruption of this loop could be a new treatment option to block the malignant progression of pancreatic cancers.

## MATERIALS AND METHODS

### Plasmid construction and virus preparation

To construct pcDNA4/SHH, the DNA fragment encoding the human *shh* gene was amplified from the cDNA of HT-29 cells using the following primer set: forward primer: 5’-TGAATTCACCATGCTGCTGCTGGCGAGATG-3’ and reverse primer: 5’-TCTCGAGGCTGGACTTGACCGCCATGCCC-3’, and inserted between the EcoRI and XhoI sites of pcDNA4/*myc*-His A (Invitrogen). To construct pcDNA4/GLI-1, the DNA fragment encoding the human *gli-1* gene was amplified from the cDNA of A549 cells using the following primer set: forward primer: 5’-TTAAAGCTTACCATGTTCAACTCGATGACC-3’ and reverse primer: 5’-TTCTAGAGGCACTAGAGTTGAGGAATTCTG-3’, and inserted between the HindIII and XbaI sites of pcDNA4/*myc*-His A. To construct pcDNA6/SMO, the DNA fragment encoding the human *smo* gene was amplified from the cDNA of MIA PaCa-2 cells using the following primer set: forward primer: 5’-TTAAAGCTTACCATGGCCGCTGCCCGCCC-3’ and reverse primer: 5’-TTCTAGAGAAGTCCGAGTCTGCATCCATG-3’, and inserted between the HindIII and XbaI sites of pcDNA6/V5-His A (Invitrogen). To construct pcDNA4/HIF-1α3A, which expresses a constitutively active mutant of HIF-1α under the control of the CMV promoter, three point mutations, P402A, P564A, and N803A, were introduced into pcDNA4/HIF-1α[25] by site-directed mutagenesis. To construct pGL3/(3’Gli-BS)_n_-Luc, the human cytomegalovirus minimal promoter and *luciferase* coding sequence between BglII and XbaI of the p5HRE-Luc plasmid [26] were first substituted for the SV40 promoter and *luciferase* coding sequence of the pGL3 Promoter Vector (Promega), respectively. The oligonucleotides containing the 3’Gli-Binding Site (3’Gli-BS) sequences (5’-CGACAAGCAGGGAACACCCAAGTAGAAGCTC-3’ and 5’-TCGAGAGCTTCTACTTGGGTGTTCCCTGCTTG-3’) [18] were annealed and multiple repeats of the resultant DNA fragments were inserted into the XhoI site of the pGL3 Promoter Vector. To construct pGEX6P-3/SHH-N (C24II), the modified N-terminal domain of the human *shh* gene, in which the N-terminal cysteine residue was substituted by two hydrophobic isoleucine residues for constitutive SHH activity [19], was amplified from the pcDNA4/SHH by PCR using the following primers: 5’-TTTGGATCCATTATTGGACCGGGCAGGGG-3’ and 5’-TCTCGAGTTAGCCTCCCGATTTGGCCGCC-3’, and was inserted between BamHI and XhoI sites of pGEX-6P-3 (GE Healthcare Bioscience). To construct the pGL3/SHH promoter, the DNA fragment of the human shh promoter region was amplified from the genome DNA of MIA PaCa-2 cells using the following primer set: forward primer: 5’-TTTAGATCTTCTGTGCTTGATGACTGAAGC-3’ and reverse primer: 5’-TCGCCCATGGAACTGATGACTTCCGAGCTG-3’, and inserted between the BglII and NcoI sites of the pGL3 Promoter Vector. For the IP experiment, the pGL3/SHH promoter was digested with XhoI and NcoI. To construct pLIB/HPV-18 E6-7, the DNA fragment encoding human papillomavirus type-18 (HPV-18) E6 and E7 genes with the 5’-URR domain was amplified from the genomic DNA of HeLa cells and inserted between the EcoRI and NotI sites of pLIB (Takara Bio). To prepare the retrovirus-expressing HPV-18 E6 and E7 genes, 24 hours after HEK-293-based packaging cells, EcoPack2-293 (BD Biosciences; 2 × 10^6^ cells/ϕ 100-mm dish) were transiently transfected with pLIB/HPV-18 E6-7, and the conditioned media were collected and filtered using Millex-HV 0.45 μm (Merck Millipore).

### Cell culture and reagents

Human pancreatic carcinoma cell lines, MIA PaCa-2, PANC-1, CFPAC-1, and BxPC-3, a human cervical epithelial adenocarcinoma cell line, HeLa, a human colorectal adenocarcinoma cell line, HT-29, a human lung carcinoma cell line, A549, and a mouse embryonic fibroblast cell line, NIH3T3, were purchased from the American Type Culture Collection. A pancreatic carcinoma cell line, SUIT-2, was purchased from the Japanese Collection of Research Bioresources Cell Bank. Mouse embryonic fibroblasts (MEFs) were immortalized by infection with retrovirus-expressing HPV-18 E6 and E7 genes, as described previously [27]. Cells were maintained in 10% FBS-containing Dulbecco's modified Eagle's medium (DMEM) supplemented with penicillin (100 units/mL) and streptomycin (100 μg/mL). Cells were incubated in a well-humidified incubator with 5% CO_2_ and 95% air at 37°C for normoxic incubation. Cells were incubated in the Bactron Anaerobic Chamber, BACTLITE-2 (Sheldon Manufacturing) or in the RUSKIN INVIVO_2_ 500 (Ruskinn) for hypoxic incubation at < 0.1% O_2_. Double-stranded RNAs for the transient silencing of HIF-1α (silencer Select Validated siRNA, HSS104774, HSS104775, HSS179231) and for the negative control (Cat# 12935-300) were purchased from Life Technologies. TAK-441 was provided by Takeda Pharmaceuticals (Fujisawa, Japan).

### Preparation of SHH-N (C24II) protein

To purify the recombinant SHH-N (C24II) protein, the pGEX6P-3/SHH-N (C24II) plasmid was transformed into BL21 (DE3) pLysS (Novagen), and the proteins were purified using Glutathione Sepharose 4B and PreScission Protease (GE Healthcare Bioscience) according to the manufacturer's instructions, as described previously [28].

### Transient transfection

Cells were transiently transfected with the indicated plasmids using the Polyfection transfection reagent (Qiagen Inc.) or Lipofectamine LTX Reagent (Life Technologies) according to the manufacturer's instructions. The cells were then cultured for 24 hours and subjected to each *in vitro* experiment (see each figure legend for details).

### Luciferase assay and western blotting

Cells were transfected with the indicated combinations of plasmids in a 24-well plate (4 × 10^4^ cells per well) and cultured in serum-free medium (for luciferase assay), or were transfected with or without the indicated plasmids in a 6-well plate (2 × 10^5^ cells per well) and cultured in 0.5% FBS-containing medium (for Western blotting) (see each figure legend for details). After culturing the cells for the indicated times, they were harvested with 100 μL of Passive Lysis Buffer (Promega) and 100 μL of Cell Lytic Buffer (Sigma-Aldrich) for the luciferase assay and Western blotting, respectively. The culture media were collected, centrifuged at 1,600 rpm for 5 minutes to remove cell debris, and then subjected to the luciferase assay and Western blotting to quantify the secreted SHH. After anti-GAL4 (DBD) rabbit polyclonal antibody (200 μg/mL; Santa Cruz Biotechnology, Cat# sc-577) as a control antibody or anti-human SHH rabbit polyclonal antibody (200 μg/mL; Santa Cruz Biotechnology, Cat# sc-9024) as an SHH-neutralizing antibody was incubated in the harvested culture media at 37°C for 1 hour, cells were cultured in the resultant culture media and subjected to the luciferase assay. In order to detect the small amount of secreted SHH proteins, which were expressed by the endogenous *shh* gene, the culture media were 10-times concentrated through centrifugation, at 14,000 x g using Amicon Ultra 0.5 mL Centrifugal Filters and Ultracel-10K (Merck Millipore) according to the manufacturer's instructions.

The luciferase assay using pRL-CMV (Promega) as an internal control to calculate the relative luciferase assay was performed using the dual luciferase assay kit according to the manufacturer's instructions (Promega), as described previously [25]. Western blotting was performed using anti-human SHH rabbit polyclonal antibody (500-fold dilution; Santa Cruz Biotechnology, Cat# sc-9024), anti-human HIF-1α mouse monoclonal antibody (500-fold dilution; BD Bioscience, Cat# 610959), anti-myc epitope tag mouse monoclonal antibody (2,500-fold dilution; Invitrogen, Cat# 46-0603), anti-V5 epitope tag mouse monoclonal antibody (1,000-fold dilution; Invitrogen, Cat# 46-0705), and anti-human β-actin mouse monoclonal antibody (500-fold dilution; BioVision or Santa Cruz Biotechnology, Cat# sc-69879) as the primary antibodies, anti-mouse and anti-rabbit immunoglobulin G horseradish peroxidase-linked whole antibodies (5,000-fold dilution; GE Healthcare Bioscience, Cat# NA931V and NA934V, respectively) as the secondary antibodies, and ECL Prime Western Blotting Detection (GE Healthcare, Cat# RPN2232) for detection according to the manufacturer's instructions.

### Quantitative real-time PCR

Quantitative real-time PCR analyses were conducted using the Thermal Cycler Dice Real Time System Single (TP-850; Takara Bio) with the SYBR Premix Ex Taq Kit (Takara Bio) and commercial primers (TaKaRa Primer Set ID HA198516 for human SHH mRNA, HA067803 for human β-actin mRNA, MA108100 for mouse bcl-2 mRNA, and MA169281 for mouse cyclin D1 mRNA) according to the manufacturer's instructions, as described previously [25, 27].

### Immunoprecipitation (IP)-qPCR experiment

Twenty-four hours after cells (1.0 × 10^6^ cells/dish in a φ100-mm dish) had been transfected with the indicated plasmids, they were treated with PBS (−) containing 1% PFA at room temperature for 10 min for crosslinkage between DNA and protein, was added with glycine for quenching (final conc. = 136 mM), and harvested with 300 μL of SDS Lysis buffer (50 mM Tris-HCl pH 8.0, 1% SDS, and 10 mM EDTA). The lysates were centrifuged at 16,100 g, and the resultant supernatants were diluted with the following dilution buffer (16.7 mM Tris-HCl pH 8.0, 1.2 mM EDTA, 167 mM NaCl, 1.1% Triton X-100, and 0.01% SDS). IP was performed using either anti-HIF-1α antibody (Abcam Cat#67) or its isotype control (Abcam Cat# 18413), and the Dynabeads Protein G Immunoprecipitation Kit (ThermoFisher). Precipitates were sequentially washed twice using each of the following buffers: Low salt buffer (20 mM Tris-HCl pH 8.0, 2 mM EDTA, 150 mM NaCl, 1% Triton X-100, and 0.1% SDS), High salt buffer (20 mM Tris-HCl pH 8.0, 2 mM EDTA, 500 mM NaCl, 1% Triton X-100, and 0.1% SDS), LiCl wash buffer (10 mM Tris-HCl pH 8.0, 1 mM EDTA, 0.25 M LiCl, 1% IGEPAL-CA630, and 1% sodium deoxycholate), and TE buffer. In order to purify DNA, the samples were first incubated at 65°C for 5 hours to drive the de-crosslinking reaction, and DNA was extracted with the QIAquick PCR purification kit (Qiagen). The precipitated DNA levels were quantified using the qRT-PCR technique as described above using the following primers: Forward: 5’-AATCCTTCAGGTGAAAATAAAGC-3’, Reverse: 5’-GAGAGGCTGCCTTTAGCAC-3’, as described previously [29].

### Proliferation assay using SF reagent *in vitro*

To examine the effect of SHH-N (C24II) on the proliferation of cultured cells *in vitro*, twenty-four hours after cells were cultured in serum-free medium with or without SHH-N (C24II) protein (3 μg/mL), they were reseeded on a 96-well plate (4 × 10^3^ cells per well) and treated with SHH-N (C24II) protein at the indicated concentration in 1% FBS-containing medium for 72 hours. To examine the effect of conditioned media on proliferation, cells were seeded on a 96-well plate (3 × 10^3^ cells per well) and cultured in 1% FBS-containing conditioned medium for 96 hours. The growth of cells was monitored with Cell Count Reagent SF (Nacalai Tesque) according to the manufacturer's instructions.

### Proliferation assay using renilla luciferase *in vitro*

To examine the influence of the existence of pancreatic cancer cells on the growth of the cocultured fibroblasts, both MIA PaCa-2 cells transfected with the SHH-expressing vector or its empty vector and fibroblasts transfected with the plasmid constitutively expressing Renilla luciferase were seeded into the same well of a 24-well plate (1 × 10^4^ cells per well, respectively), and cocultured in 1% FBS-containing culture medium under normoxic or hypoxic conditions for 24 hours. Cell lysates were harvested with 100 μL of Passive Lysis Buffer, and the growth of fibroblast cells was monitored as the intensity of Renilla luciferase bioluminescence using the STOP & Glow solution of the dual luciferase assay kit according to the manufacturer's instructions.

### Statistical analyses

The significance of differences was determined using Student's *t*-test. A *P*-value < 0.05 was considered to be significant.

## SUPPLEMENTARY MATERIALS FIGURES


