# Supplementary material for: HIF-1 maintains a functional relationship between pancreatic cancer cells and stromal fibroblasts by upregulating expression and secretion of Sonic hedgehog
Source: Oncotarget. 2018 Jan 11;9(12):10525–35. doi: 10.18632/oncotarget.24156 (PMC5828220; doi:10.18632/oncotarget.24156)
Supplement: Supplementary file 1 [file oncotarget-09-10525-s001.pdf]

HIF-1 maintains a functional relationship between pancreatic cancer cells and stromal fibroblasts by upregulating expression and secretion of Sonic hedgehog

SUPPLEMENTARY MATERIALS

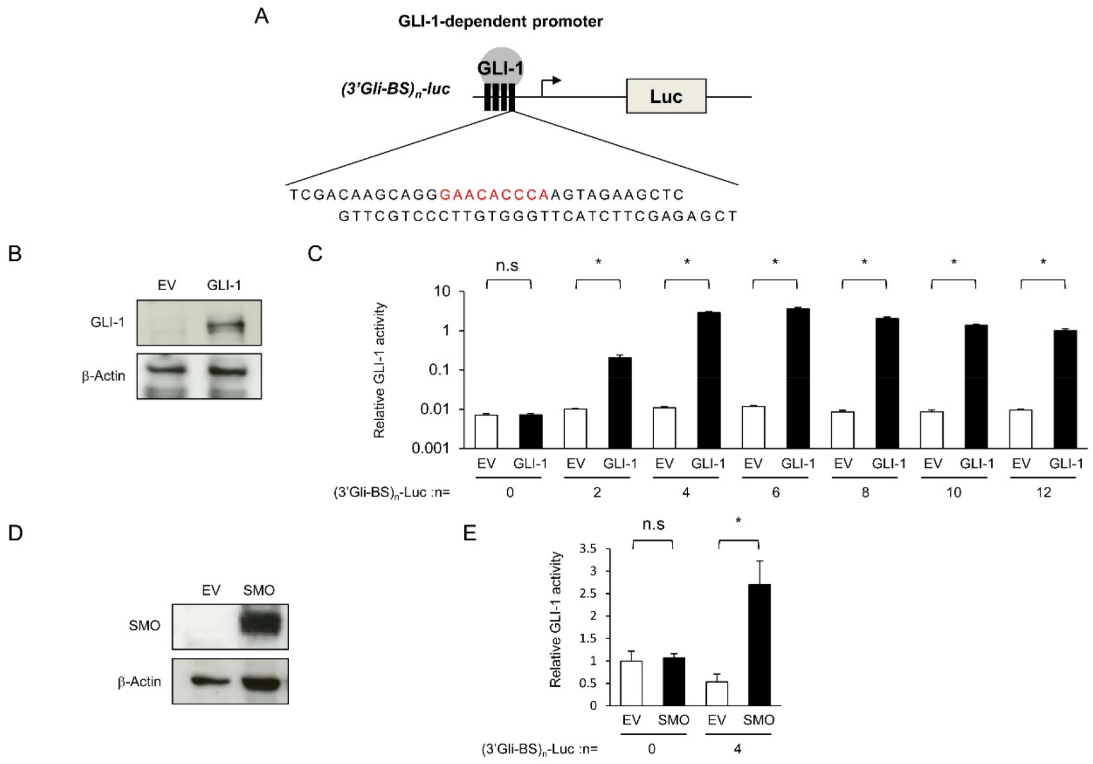

Supplementary Figure 1: Establishment of a system to monitor the hedgehog signaling activity. (A) Schematic diagram of the *(3'Gli-BS)$_n$*-luc reporter gene. **(B)** NIH3T3 cells were transiently transfected with either pcDNA4/GLI-1 (GLI-1) or pcDNA4/*myc*-His A (EV), and cell lysates were subjected to Western blotting using the indicated antibodies. **(C)** NIH3T3 cells were transiently co-transfected with either pcDNA4/GLI-1 (GLI-1) or pcDNA4/*myc*-His A (EV), and the *(3'Gli-BS)$_n$*-luc reporter vector containing the indicated number of the 3'Gli-BS, and subjected to the luciferase assay. **(D)** NIH3T3 cells were transiently transfected with either pcDNA6/SMO (SMO) or pcDNA6/V5-His A (EV), and cell lysates were subjected to Western blotting using the indicated antibodies. **(E)** MEFs were transiently co-transfected with either pcDNA6/SMO (SMO) or pcDNA6/V5-His A (EV), and either pGL3/(3'Gli-BS)0-Luc or pGL3/(3'Gli-BS)$_4$-Luc, and subjected to the luciferase assay.

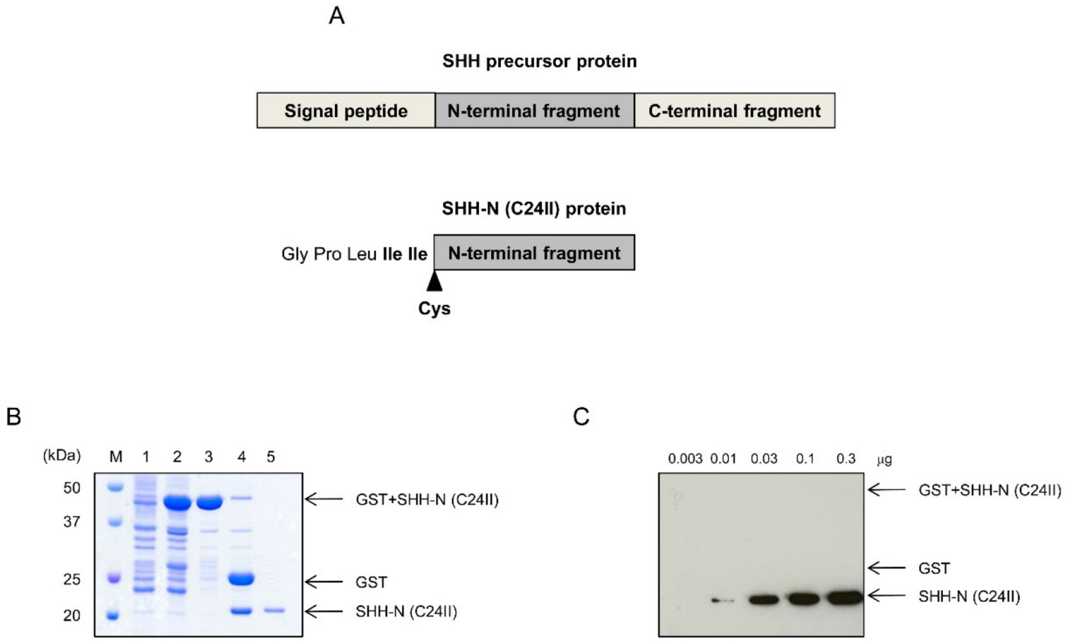

Supplementary Figure 2: Purification of the recombinant SHH protein. **(A)** Schematic diagram of the recombinant SHH-N (C24II) protein. **(B)** SDS-PAGE was conducted to confirm the purity of the recombinant SHH-N (C24II) protein. Lane 1, total lysates before IPTG induction; Lane 2, total lysates after IPTG induction; Lane 3, proteins in cell lysate captured by the Glutathione Sepharose 4B beads before PreScission Protease cleavage; Lane 4, proteins in cell lysate captured by the Glutathione Sepharose 4B beads after PreScission Protease cleavage; Lane 5, supernatants from Glutathione Sepharose 4B beads (purified recombinant protein); M, protein marker. **(C)** The indicated amounts of the recombinant SHH-N (C24II) protein were subjected to Western blotting using anti-SHH Ab.
